# MCAD deficiency caused by compound heterozygous pathogenic variants in *ACADM*

**DOI:** 10.1038/s41439-021-00177-3

**Published:** 2022-01-17

**Authors:** Fumikatsu Nohara, Go Tajima, Hideo Sasai, Yoshio Makita

**Affiliations:** 1 Department of Pediatrics, Asahikawa-Kosei General Hospital, Hokkaido, Japan; 2 Division of Neonatal Screening, Research Institute, National Center for Child Health and Development, Tokyo, Japan; 3 Department of Pediatrics, Graduate School of Medicine, Gifu University, Gifu, Japan; 4 Department of Genetic Counseling, Asahikawa Medical University Hospital, Hokkaido, Japan

**Keywords:** Metabolic disorders, Disease genetics

## Abstract

Medium-chain acyl-coenzyme A dehydrogenase (MCAD) deficiency is an autosomal recessive disease caused by biallelic pathogenic *ACADM* variants. We report a case of an asymptomatic Japanese girl with MCAD deficiency caused by compound heterozygous pathogenic variants (NM_000016.5:c.1040G > T (p.Gly347Val) and c.449_452delCTGA (p.Thr150ArgfsTer4)). Because the MCAD residual activity in lymphocytes of the patient was below the limit of quantification, both variants are likely to cause complete loss of MCAD enzymatic activity.

Medium-chain acyl-coenzyme A dehydrogenase (MCAD, EC 3.1.2.20) deficiency (OMIM 201450) is the most common disorder of fatty acid beta-oxidation caused by biallelic pathogenic *ACADM* gene (OMIM 607008) variants. In hepatocytes, fatty acid oxidation provides acetyl-CoA for hepatic ketogenesis, which serves as a major source of energy once hepatic glycogen stores are depleted. Because MCAD catalyzes the first step of mitochondrial beta-oxidation for medium-chain acyl-CoAs, a previously healthy individual with MCAD deficiency (MCADD) suddenly experiences symptoms triggered by periods of fasting or illness, such as viral infection. Symptoms include hypoketotic hypoglycemia and vomiting, which may quickly progress to lethargy, seizure, and coma. Sudden unexpected death in infancy (SUDI) has also been recognized as the first manifestation of MCADD^1,2^. It is reported that before newborn screening (NBS) was available, 20–25% of affected infants and young children died suddenly during the first episode of metabolic decompensation and that severe neurological sequelae were often observed in survivors^3^. In contrast, once the diagnosis is established, implementation of frequent feeding to avoid any prolonged fasting and appropriate management of periods of illness can be expected to almost completely prevent onset of the disease^4,5^.

The prevalence of MCADD varies across ethnic groups. In Japan, it is estimated to be approximately 1 in 100,000 births^6,7^, which is considerably lower than that in Western countries. In 2014, Japan initiated a nationwide NBS for MCADD using tandem mass spectrometry (MS/MS), which facilitated the identification of many patients and novel variants. Although it is important to evaluate the risk of acute metabolic decompensation for each variant, a lack of knowledge about the association between variants and enzyme activity makes it difficult to infer phenotype from genotype. Here, we report a Japanese girl with MCADD with an unknown pathogenicity *ACDM* gene variant.

The patient was the second child of healthy and nonconsanguineous parents. Her family, including her 4-year-old brother, had no history of metabolic disorders. She was born at 39 weeks of gestation by spontaneous vaginal delivery without neonatal asphyxia. At birth, her weight was 2775 g (−0.55 SD). She was discharged from the maternity clinic at 5 days of age, with no special remarks regarding her perinatal history. Because elevated octanoylcarnitine (C8) levels and C8/decanoylcarnitine (C10) ratios (C8/C10 ratios) were noted by NBS, she was referred to our hospital. At the first visit, no abnormalities were observed on physical, neurological, or blood examination. Results of NBS at 5 days of life were 39.453 nmol/ml, 4.027 nmol/ml (cutoff value >0.3 nmol/ml), and 8.628 (cutoff value >1.4) for free carnitine (C0), C8, and C8/C10 ratios, respectively. Urinary organic acid analysis at 7 days of life detected 2.9 nmol/ml hexanoylglycine (cutoff value >0.5 nmol/ml) and 12.4 nmol/ml suberylglycine (cutoff value >0.5 nmol/ml). Although the findings of urinary organic acid analysis in this disease are not specific and serum acylcarnitine analysis is recommended as a basis for diagnosis, serum acylcarnitine analysis was not feasible in our hospital. Therefore, we suspected MCADD based on the results of acylcarnitine analysis of dried blood spot samples and urinary organic acid analysis and performed enzyme activity analysis and genetic analysis for a definitive diagnosis. After written informed consent was obtained from her parents, next-generation sequencing (NGS) of the patient’s *ACADM* gene was performed, and we identified two heterozygous mutations: NM_000016.5:c.449_452delCTGA (p.Thr150ArgfsTer4) and c.1040G > T (p.Gly347Val). Targeted Sanger sequencing identified c.449_452delCTGA and c.1040G > T in her father and mother, respectively (Fig. 1). c.449_452delCTGA has been reported to be the most common pathogenic variant in the Japanese population^7,8^. c.1040G > T is not listed in public databases, such as the Exome Variant Server (http://evs.gs.washington.edu/EVS/), 1000 Genome Database (http://browser.1000genomes.org/), dbSNP (http://www.ncbi.nlm.nih.gov/snp/), Genome Aggregation Database (GenomAD, https://gnomad.broadinstitute.org/), Human Genetic Variation Database (HGVD, http://www.hgvd.genome.med.kyoto-u.ac.jp/), and Japanese Multi Omics Reference Panel (jMORP, https://jmorp.megabank.tohoku.ac.jp/201909/). Moreover, this missense variant is predicted to be probably damaging, with a score of 0.979, by PolyPhen2 (http://genetics.bwh.harvard.edu/pph2/) and deleterious, with scores of −8.147 and −5.25173, by PROVEAN (http://provean.jcvi.org/index.php/) and PANTHER (http://www.pantherdb.org/tools/csnpScoreForm.jsp/), respectively. Although c.1040G > T has very recently been reported as a novel variant observed in 1 of 24 Chinese patients with MCADD^9^, no detailed assessment was performed to classify the pathogenicity of this variant with evidence according to the American College of Medical Genetics and Genomics and the Association for Molecular Pathology (ACMG/AMP) guidelines^10^. This missense variant is absent in the population database (PM2); it may cause a deleterious effect on the gene product, as supported by multiple lines of computational evidence (PP3). It is detected in *trans* with a pathogenic variant (PM3) and was observed in the gene specifically associated with the patient’s phenotype (PP4). Consequently, this variant is classified as ‘likely pathogenic’ according to ACMG guidelines^10^. We measured the enzymatic activity of MCAD using the patient’s lymphocytes^11^, and residual activity was 0% of normal (below the limit of quantification); thus, both variants appear to cause complete loss of MCAD function (Fig. 2). The c.449_452delCTGA variant has been reported as a pathogenic variant that abolishes enzyme activity^6,7^, and the c.1040G > T variant is also predicted to severely impair enzyme activity. It is difficult to estimate the severity of the disease from the genotype, yet the lack of MCAD enzymatic activity in the patient indicates that both variants are associated with a high risk of metabolic decompensation. At 21 months of age, the patient experienced no episodes of metabolic decompensation without any medication, and her physique and development were normal.

**Fig. 1:**
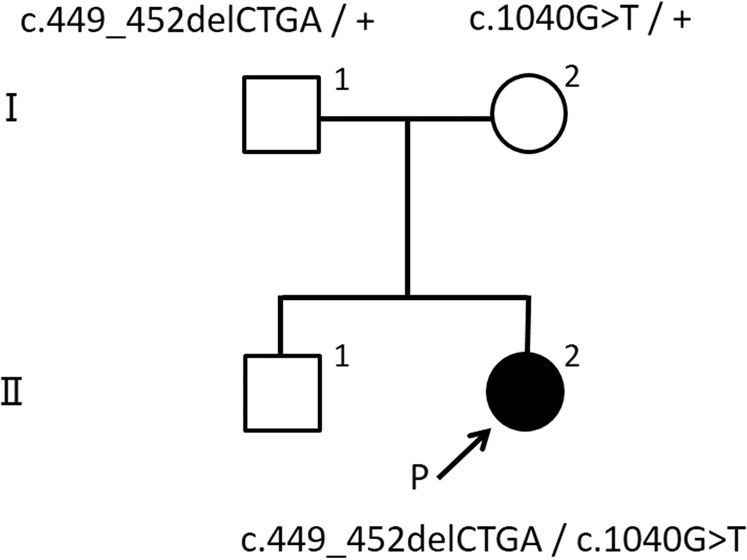
Pedigree of the family with MCADM genotypes. Two heterozygous mutations were identified in the proband (arrows), and each mutation was identified heterozygously in father and mother, respectively.

**Fig. 2:**
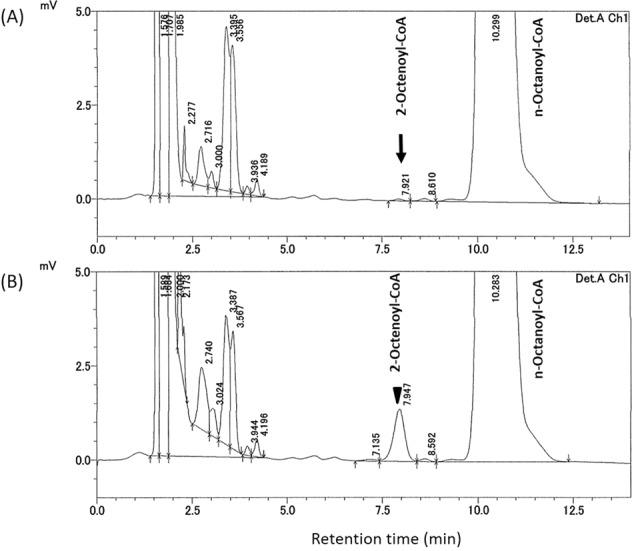
Chromatogram of the assay for MCAD activity in lymphocytes. MCAD activity was determined by 2-octenoyl-CoA production using high-performance liquid chromatography, as previously described^11^. **A** The MCAD residual activity of the proband was below the limit of quantification (arrow). **B** Normal control (arrowhead).

In conclusion, we identified a pathogenic *ACADM* gene missense variant, NM_000016.5:c.1040G > T, in a patient with NBS-positive asymptomatic MCADD. The complete loss of MCAD activity in the patient’s lymphocytes indicates that those with compound heterozygous c.1040G > T (p.Gly347Val) and c.449_452delCTGA (p.Thr150ArgfsTer4) mutations are at high risk of metabolic decompensation.

## HGV database

The relevant data from this Data Report are hosted at the Human Genome Variation Database at 10.6084/m9.figshare.hgv.3112.
